# Supplementary material for: Energy-ordered resource stratification as an agnostic signature of life
Source: Nat Commun. 2025 Mar 28;16:2867. doi: 10.1038/s41467-025-58206-6 (PMC11953477; doi:10.1038/s41467-025-58206-6)
Supplement: Supplementary file 1 — Supplementary Information [file 41467_2025_58206_MOESM1_ESM.pdf]

Supplementary information
Energy-ordered resource stratification as an agnostic signature of life

Akshit Goyal and Mikhail Tikhonov

VARIANTS OF THE MODEL TESTED

Supplementary Note 1: Simulating cross-feeding

We simulated a variant of our model with cross-feeding by numerically evolving the following equations

$$\frac{\partial N_i(x,t)}{\partial t} = N_i(x,t)\Big(\gamma(1-\ell)g_i(\vec{N},\vec{R}) - \sum_{j\neq i} A_{ij}N_j(x,t)\Big) + D_N\nabla^2 N_i(x,t), \tag{1}$$

$$\frac{\partial R_\alpha(x,t)}{\partial t} = -\sum_i f_{i\alpha}(\vec{N},\vec{R}) + \sum_\beta \ell D_{\alpha\beta} f_{i\beta}(\vec{N},\vec{R}) + D_R\nabla^2 R_\alpha(x,t), \tag{2}$$

where

$$g_i(\vec{N},\vec{R}) = \sum_{\alpha=1}^{M} Y_\alpha k_{i\alpha} R_\alpha(x,t) - m_i, \tag{3}$$

$$f_{i\alpha}(\vec{N},\vec{R}) = k_{i\alpha}R_\alpha(x,t)N_i(x,t), \tag{4}$$

$\ell = 0.5$ is a leakage parameter that controls what fraction of consumed resources are secreted as metabolic byproducts, and $D_{\alpha\beta}$ is a cross-feeding matrix that encodes which resources α can be produced from which others β as byproducts. For simplicity, we assumed that D mimicked a linear chain: consumption of resource 1 released resource 2; consumption of resource 2 released resource 3; and so on. Similar to the main text, all simulations were done on a 1D domain of length $x \in [0, L]$ where $L = 100$ in arbitrary units, assuming no boundary flux Neumann boundary conditions for species. In contrast with the main text, we assumed that only resource 1 was externally supplied, entering the system at $x = 0$ at flux K; all other resources were internally generated via cross-feeding. No resource could escape the system, resulting in no flux boundary conditions at $x = L$ for all resources; and at $x = 0$ for all cross-fed resources. For all simulations, we set $D_N = 10$, $D_R = 20$, $m_i = m = 0.1$ and $k_{i\alpha} = 1$ for all species and resources. We separately verified that small variations in these parameters, which break their degeneracy, do not affect our conclusions (simulations not shown). For each simulation with $M = 3$ resources and $S = M = 3$ species, we chose the energy content Y_α of each resource α as $Y_1 = 1.8, Y_2 = 1.3, Y_3 = 1$. For competitive interaction strengths A_{ij} between species i and j, we picked them randomly from a normal distribution with mean 0.4 and standard deviation 0.1. All diagonal entries $A_{ii} = 0$ were left out of this procedure. For initial conditions, we set homogeneous initial conditions for species, while choosing quartically decaying profiles for resources, numerically obtained to satisfy the boundary conditions. A representative example of the long-time species and resource profiles is plotted in Fig. S1. As in the original model, we observe that energy-ordered resource stratification emerges even when only one resource is provided externally, as metabolic byproducts always have lower energy content and can only be depleted once they have diffused sufficiently away from the species producing them.

Supplementary Note 2: Including concentration-dependent growth rates & ecological redundancy

In our original model, we assumed that the growth (self-replication) rate of all species increases linearly with local resource concentration. While simple, this assumption might not hold when the local resource concentration is large. Moreover, it is common for the same resource to be consumed by multiple strains, some of which grow better at higher resource concentrations, while others grow better at lower concentrations (allowing them to coexist). To test whether such resource-dependence affects our conclusions about energy-ordered resource stratification, we simulated a variant

of our model with the same equations (1) and (2), but with per-capita growth rates g_i having the commonly assumed saturating form

$$g_i(\vec{N}, \vec{R}) = \sum_{\alpha=1}^{M} Y_\alpha \frac{k_{i\alpha} R_\alpha(x,t)}{K_{i\alpha} + R_\alpha(x,t)} - m_i. \tag{5}$$

To simulate an example of the consequences of changing these model assumptions, we first took the simple case of 3 species on 2 resources (Fig. S2a): 2 of which (blue) grew by consuming the same high-energy content resource (blue triangle), while the remaining (orange) consumed the second lower-energy content resource (orange triangle). For the blue species using the same resource, we assumed a trade-off in their maximal consumption rate $k_{i\alpha}$ and half-saturating resource concentration $K_{i\alpha}$ (Fig. S2b). We simulated this model using the same parameters and initial conditions as the original model in the main text, and observed that the two resources stratified in decreasing order of their energy content (Fig. S2d). The species also stratified in space, but there was significant overlap between two species: one that grew better at lower concentrations of the high-energy resource (light blue) and the one that consumed the low-energy resource (orange; Fig. S2c). This suggested two statements: (1) that energy-ordered resource stratification was robust to the concentration-dependent growth assumed, and (2) that resource stratification was stronger than species stratification.

To systematically test these two statements, we repeated our simulations for a more general case of 8 randomly generated species growing on 5 resources. The 3 resources with highest energy content each had two species capable of consuming them, one better at high concentration and the other at low concentration. The last 2 resources could only be consumed by 1 species each, and grew well at low concentrations ($K_{i\alpha} = 10^{-3}$). We quantified the resource stratification order parameter as in the main text, by computing the penetration depth of each resource (Fig. S2f). To quantify the depth of species, we computed the expectation value of their position, i.e., the mean position using their species abundance profiles (Fig. S2e). A representative example is shown in Fig. S2g–h. We observed that our two statements indeed generalized to more species and resources. Importantly, we concluded that resources were still stratified in decreasing order of their energy content. Further, resource stratification was slightly stronger (stratification order parameter 0.9) than species stratification (stratification 0.7).

Supplementary Note 3: Varying the degree of generalism

In our original model, each biological species could exclusively consume 1 resource. To test if we would still observe stratification if species were instead generalists, i.e., allowed to consume more than 1 resource, we simulated a variant of our model where we could vary the degree of generalism. In this variant, we simulated our original model (Eqns (1)–(4)), with the only change being that the resource consumption matrix $k_{i\alpha}$ was not a diagonal matrix any more. Instead, we set all off-diagonal entries — corresponding to species consuming all resources — to a value Δ indicating the degree of generalism. We kept the diagonal entries at 1 in accordance with the model in the main text. In the original model $\Delta = 0$. For each value of Δ between 0 and 1 that we tested, we simulated 10 randomly generated ecosystems, in each case measuring the resource stratification order parameter. As we increased Δ and all species became generalists, we found that the stratification order parameter at first remained close to 1 (Fig. S3). This suggested that energy-ordered resource stratification was robust to including generalists. If Δ was large and comparable to the diagonal entries of the consumption matrix $k_{i\alpha}$, this would imply a situation where all species were statistically indistinguishable in terms of their preferred energy-providing resource. In this situation, we would not expect resource stratification, and this was indeed what we observed (Fig. S3). Indeed, we found that beyond a certain degree of generalism $\Delta \approx 0.4$, resources ceased to be stratified.

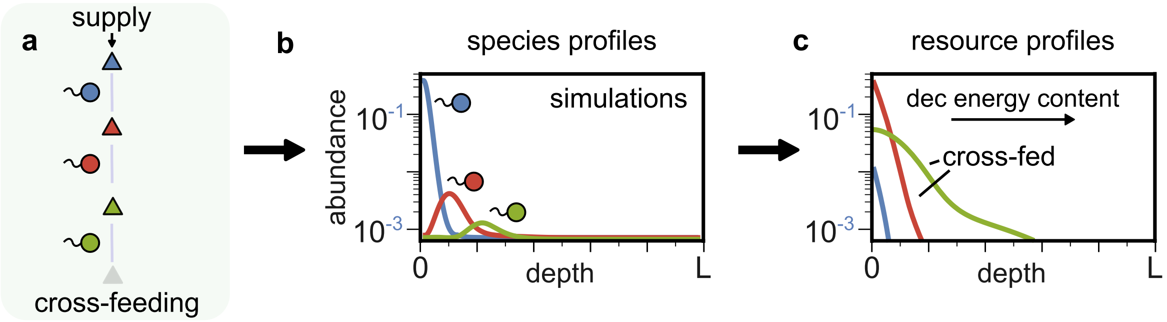

FIG. S1. **Energy-ordered resource stratification also emerges from cross-feeding — when only one resource is supplied and the others are internally generated as byproducts.** Simulating a minimal model incorporating self-replication, ecology — via both antagonism and cross-feeding (see supplementary text for details) — shows that cross-feeding also supports the emergence of spatially stratified profiles of (b) species and (c) resources. Shown here is an example from a simulation for 3 species and 3 resources (triangles; the last, grey resource acts as a "waste" and is not shown). Only the blue resource is supplied externally. As the blue species consumes this resource, it secretes the red resource, which the red species consumes, secreting the green resource, and so on.

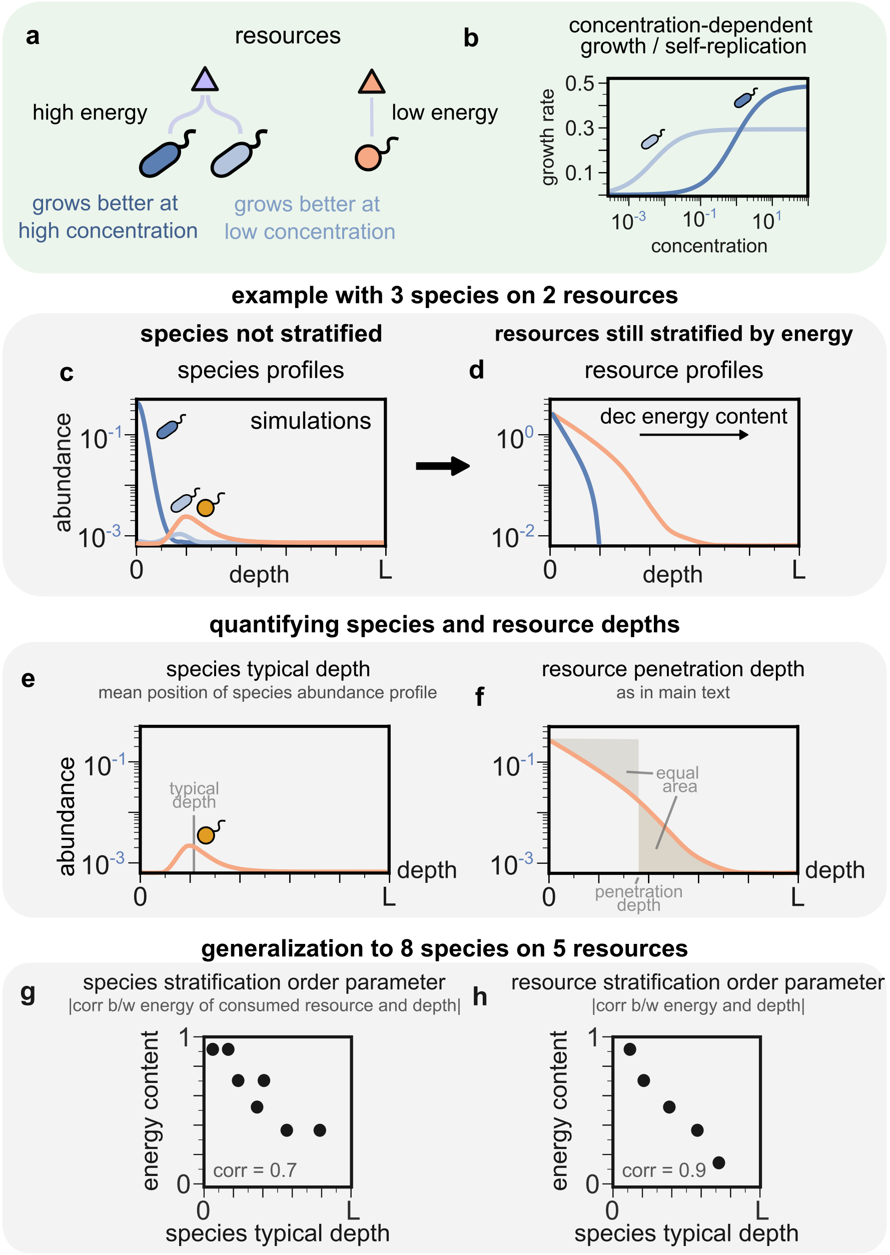

FIG. S2. **Energy-ordered resource stratification is robust to concentration-dependent growth & ecological redundancy.** (a) Schematic showing 3 species (squiggles) growing on 2 resources (blue and orange triangles) for a variant of our model with concentration-dependent growth modeled via Monod, or Michaelis-Menten kinetics (see supplementary text for details). In this example, the two species that both consume the high-energy blue resource grow better at high and low concentrations of this resource, respectively; the orange species grows well at high concentrations of the low-energy orange resource. (b) Growth rate profiles for the two blue species in (a) as a function of the concentration of the high-energy resource (blue triangle). Both species show a trade-off in their ability to grow well at high versus low concentrations of the resource. (c–d) Simulations of the model variant in (a) and (b) still show that (d) the two resources stratify by energy content while (c) species may be less stratified (e.g., the light blue and orange species appear weakly stratified). This model variant breaks the competitive exclusion principle by allowing species to coexist on fewer resources via the assumed trade-off in concentration-dependent growth. (e) We quantifying the typical depth of species by measuring their average abundance-weighted position given by their simulated profile (example shown). (f) We quantify the resource penetration depth akin to the main text. (g–h) show scatter plots of the the species and resource depths with their corresponding energy content for another representative example with 8 species growing on 5 resources (see supplementary text for details); for species we use the energy content of the resource they consume. One of the 8 species simulated eventually went extinct. The correlation between these quantities provides a stratification order parameter, which shows that — even in this model variant — resources remain strongly stratified by energy content.

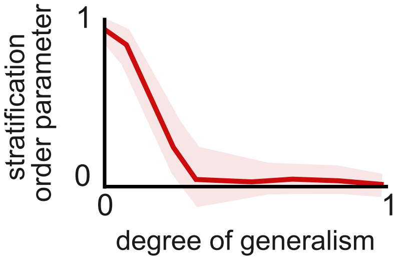

FIG. S3. **As species use multiple resources with decreasing specificity of preference, stratification decreases.** Plot showing the resource stratification order parameter (measured as shown in Fig. 3a) as a function of the degree of generalism Δ in species for 10 randomly generated ecosystems with $S = 5$ species and $M = 5$ resources. In this variant of our model, we allow species to consume all resources—i.e., to become generalists—by introducing a fixed off-diagonal term Δ in the consumption matrix $k_{i\alpha}$ (see supplementary text for details). For small Δ, energy-ordered stratification persists. As Δ increases, energy-ordered stratification signature eventually disappears due to the increasing similarity between all biological species. Note that for $\Delta > \frac{1}{M-1} = 0.25$, species obtain more energy from other resources than the one they "specialize" on. Values plotted indicate the mean resource stratification order parameter over 10 randomly generated systems for each value of Δ, while the shaded intervals indicate the standard error in the mean.
